# A modular paper-and-plastic device for tuberculosis nucleic acid amplification testing in limited-resource settings

**DOI:** 10.1038/s41598-019-51873-8

**Published:** 2019-10-25

**Authors:** Navjot Kaur, Joy S. Michael, Bhushan J. Toley

**Affiliations:** 1 Department of Chemical Engineering, Indian Institute of Science, 560012 Bangalore, India; 2 Department of Microbiology, Christian Medical College, 632004 Vellore, India

**Keywords:** Genetic testing, Biomedical engineering

## Abstract

We present a prototype for conducting rapid, inexpensive and point-of-care-compatible nucleic acid amplification tests (NAATs) for tuberculosis (TB). The fluorescent isothermal paper-and-plastic NAAT (FLIPP-NAAT) uses paper-based loop mediated isothermal amplification (LAMP) for DNA detection. The cost of materials required to build a 12-test-zone device is $0.88 and the cost of reagents per reaction is $0.43. An inexpensive imaging platform enables filter-free fluorescence detection of amplified DNA using a cell-phone camera. FLIPP-NAAT can be operated by an untrained user and only requires a regular laboratory incubator as ancillary equipment. All reagents can be dry-stored in the device, facilitating storage and transportation without cold chains. The device design is modular and the assay demonstrated high specificity to Mycobacterium tuberculosis (*Mtb*), analytical sensitivity of the order of 10 copies of *Mtb* gDNA, and tolerance to complex samples. The clinical sensitivity and specificity of sputum-based FLIPP NAAT tests were 100% (zero false negatives) and 68.75% (five false positives), respectively (N = 30), using Xpert MTB/RIF assay as the reference standard. FLIPP-NAAT has the potential to provide affordable and accessible molecular diagnostics for TB in low- and middle-income countries, when used in conjunction with an appropriate sample preparation technique. Although demonstrated for the detection of TB, FLIPP-NAAT is a platform technology for amplification of any nucleic acid sequence.

## Introduction

Tuberculosis (TB) is the deadliest infectious disease today, having claimed over 1.6 million lives worldwide in 2017^1^. A lack of rapid and inexpensive TB diagnostic tests has been a major roadblock in managing the disease. Because the disease primarily affects people from poor economic backgrounds, it is imperative to provide access to state-of-the-art rapid TB diagnostic technologies to these populations. Culture tests, the gold standard for definitive TB diagnosis, take a long time (6 to 8 weeks) and are associated with other challenges^2^. Nucleic acid amplification tests (NAATs) are the new gold standards for rapid TB diagnosis.

The WHO-endorsed GeneXpert (Xpert MTB/RIF assay) provides an unprecedented level of automation of the multiple steps involved in a NAAT and enables a minimally-trained user to conduct a NAAT, but the cost of this instrument remains prohibitively high in many settings^3^. Another PCR-based test from a joint venture of Molbio diagnostics and Bigtec labs (Bangalore, India)^4^ consists of two units -Trueprep Mag for sample preparation and Truelab Uno for DNA amplification. This device consists of one additional step compared to the Xpert MTB/RIF assay because purified DNA generated by the Trueprep Mag must be manually transferred into the Truelab Uno unit. Compared to Xpert MTB/RIF assay, this test is only slightly lower in terms of user-friendliness, but moderately lower in terms of instrument-cost. On the low end of the cost spectrum, there are generic PCR machines like the Open qPCR (CHAI, Santa Clara, CA), not specifically designed for TB NAATs, and are only suitable for research labs for operation by skilled users. Instrument costs have also been driven down by isothermal nucleic acid amplification methods^5,6^ replacing PCR, a strategy which has recently gained significant interest for POC diagnostics. A commercially available TB testing kit and instrument from Eiken (Tokyo, Japan), based on an isothermal NAAT, have been endorsed by the WHO^7^. While the instrument cost is low, conducting the assay involves multiple careful pipetting steps that must be conducted by trained users. Hence, there continues to be a tradeoff between the cost of TB NAATs and their user-friendliness and ultimately, ease-of-use is the most critical parameter in making a NAAT amenable to use at the point-of-care. Therefore, there exists significant room for technological innovation to provide easy-to-use, yet affordable solutions for TB-NAATs.

Paper-based isothermal NAATs have recently emerged as popular platforms for low-cost nucleic acid detection. These platforms have the potential to fill the abovementioned gap^8,9^ and the opportunities and challenges in the area of paper-based NAATs have recently been extensively reviewed^10,11^. While paper-based NAATs have been demonstrated for the detection of various infectious diseases^11^, there has been only one report of their use for TB detection. Shetty *et al*.^12^ used helicase-dependent amplification (HDA) in chromatography paper for detection of TB. The target DNA was an 84-base pair fragment from the insertion sequence 6110, amplified using PCR. A disk of chromatography paper containing dried HDA reagents was rehydrated with a solution of this synthetic target, sealed in a polyethylene pouch, and kept on a heater to conduct HDA. The detection was based on end-point fluorescence using a fluorescence microscope.

In this study, we present a new paper-based device for conducting isothermal NAATs at the point-of-care and demonstrate its utility in sensitive and specific detection of TB, directly from whole Mycobacterium tuberculosis (*Mtb*) genomic DNA (gDNA). We also present a very low-cost imaging box for filter-free end-point fluorescence detection of the amplified DNA using a cell phone. To the best of our knowledge, this is the first demonstration of a paper-based NAAT for TB starting from gDNA as the template. Compared to existing paper-based NAATs for other infectious diseases, our device presents several improvements in terms of ability to multiplex, user experience, and cost reduction. The prototype called fluorescent isothermal paper-and-plastic NAAT (FLIPP-NAAT) uses loop mediated isothermal amplification (LAMP) for DNA amplification and the only ancillary equipment required for testing is a laboratory incubator. The design of the FLIPP-NAAT is ‘modular’ and the number of testing zones can easily be increased by increasing the number of modules. FLIPP-NAAT is sensitive (10 copies, analytical sensitivity) and specific towards *Mtb* gDNA and can detect DNA directly from viscous sputum. TB testing from 30 clinical samples demonstrated a clinical sensitivity of 100% and clinical specificity of 68.75%. By providing a very low-cost and user-friendly means for DNA amplification, FLIPP NAAT represents a significant step towards filling the technological gap in TB-NAATs. In combination with an appropriate DNA extraction procedure, it promises to be an essential tool for conducting NAATs in places like rural India.

## Materials and Methods

### Materials for fabrication

Standard 17 glass fiber (referred to as paper pads henceforth) was acquired from GE Healthcare Life Sciences (Bangalore, India). A double-sided pressure sensitive adhesive (PSA; 3M^TM^ 9731) was used to secure the different layers of the device together. Transparent acrylic sheets (2.8 mm-thick), black acrylic sheets (2 mm-thick) and transparency sheets (0.07 mm) were acquired locally. All materials were cut using a 50 W CO_2_ laser in a VLS 3.60 laser engraver (Universal Laser Systems, Scottsdale, AZ). All designs were created in AutoCAD (Autodesk, San Rafael, CA).

### DNA amplification in tube

*Mtb* gDNA (strain H37Rv) was obtained from BEI resources (Manassas, VA, USA) and quantified using a Nanodrop 2000 (ThermoFisher Scinetific). DNA amplification was performed using loop-mediated isothermal amplification (LAMP) targeting a region of the *hspX* gene of *Mtb* using six primers as described by Bi *et al*.^13^ (primer sequences are provided in Supplementary Table S1). Initial standardization of the LAMP reaction was done for a 25 µl reaction with the following composition: 1.6 µM forward inner primer (FIP) and backward inner primer (BIP), 0.2 µM forward outer primer (F3) and backward outer primer (B3), 1.2 µM forward loop primer (FLP) and backward loop primer (BLP), 1X LAMP reaction buffer, 8 mM MgSO_4_, 0.9 M Betaine (B0300-1VL, Sigma-Aldrich), 2.5 µl dNTP mix (10 mM each, N0447S, New England Biolabs (NEB)), 8 units of *Bst 2*.*0* WarmStart® DNA polymerase (M0538S, NEB), 1 µl of the target DNA, and DEPC water to make up the final volume to 25 µl. Specificity of the assay was tested by replacing the *Mtb* gDNA with equal volume of *Escherichia coli* gDNA, *Mycobacterium smegmatis* gDNA, or plasmid DNA containing inserts for *Hepatitis C* and *Dengue* virus. Tolerance to human gDNA was also tested by mixing 10–10^4^ starting copies of human gDNA (G3041, Promega) with 1000 and 100 starting copies of *Mtb* gDNA. A range of temperatures starting from 56 °C to 65 °C was tested to find the optimum reaction temperature. For real-time LAMP reactions, four different fluorescent dyes - SYBR green I (Sigma-Aldrich, S9430), SYBR safe (Invitrogen, P/NS33102), Evagreen (Biotium, 31019) and SYBR gold (Invitrogen, S11494) were tested using a Quant Studio 3 (Applied Biosystems).

### DNA amplification in FLIPP-NAAT

The reaction was ported from tube to paper by making the entire reaction mix in a tube along with the template and adding it to paper pads through sample addition holes. The device was then sealed and kept in an incubator set to the optimum reaction temperature for a specified period, following which, the reaction was terminated by removing the device from the incubator. Over the course of working on this device, the reaction volume was reduced to 12.5 µl maintaining the concentrations of all reagents.

### Detection of LAMP products using gel electrophoresis

LAMP products were run in 2% agarose gels (115V) stained with ethidium bromide and the gels were imaged in a custom-made gel imager. In cases where DNA amplification was done in FLIPP-NAAT, the device was carefully pried open using a blade and fluid from within the paper pads was eluted by centrifugation at 10,000 rpm for 1 minute before conducting electrophoresis. Target specific amplification was confirmed by digesting the LAMP amplicons with the restriction enzyme *AatII*. DNA ladders (O’ Gene Ruler; Low range and 100 bp) were obtained from ThermoFisher Scientific.

### Fluorescence detection of LAMP products in FLIPP-NAAT

Two fluorescent DNA intercalating dyes, SYBR Green I (Sigma- Aldrich, S9430) and SYBR Safe (Invitrogen, P/NS33102) were tested for end-point detection of LAMP amplicons. On completion of the reaction, the device was removed from the incubator, top flap was peeled, DNA intercalating fluorescent dye (5 µl, 50X) was added to the reaction pads through the top holes, and top flap was placed back to seal the device. The device was then placed inside a custom-made imaging station such that bottom transparent acrylic faced the source of excitation and the camera. The imaging station (20 cm × 22 cm) was a cuboid without top cover, made from black acrylic, and consisted of an inclined platform to fix the phone. A UV torch was used as the excitation source and fluorescent images were captured using a smartphone (Redmi 5, 64 GB Phone) with the HDR option turned off. The imaging set-up was checked for uniformity of field by adding 12.5 µl of a well-mixed LAMP amplicons and fluorescent dye mix to each of the 12 paper pads and imaging it within the box. The images were analyzed using ImageJ in the green channel and two-tailed Student’s t-tests were used for statistical comparison.

### DNA amplification from dry-stored reagents in FLIPP-NAAT

Circular paper pads were pre-treated by dipping in a 1% bovine serum albumin (BSA) and 0.1% tween20 solution for two hours, following which the excess fluid was removed by placing the pads on Kimwipes. These pre-treated paper pads were dried at 37 °C for at least 24 hours and stored at 37 °C until further use. For dry storage of LAMP reagents in paper pads, 12.5 µl of the LAMP mix containing an optimum trehalose concentration and water replacing the template was made in tubes and added to the paper pads. The paper pads were immediately put under liquid nitrogen and placed in a lyophilizer (Labconco, 7670560) set to −45 °C and 0.01mBar for 12 hours. After removal from lyophilizer, FLIPP-NAAT was assembled using these paper pads and LAMP reactions were conducted within 2 hours. Paper pads were rehydrated with 9 µl of a template solution (1 µl target DNA + 8 µl of water), the device sealed, and put inside an incubator for the reaction.

### Detection of TB from complex samples

Mock sputum was prepared by intermittently dissolving 2 g of methyl cellulose (Sigma-Aldrich, M0262) in 90 ml of autoclaved distilled water with constant stirring at 80 °C. This solution was then stored at 42 °C overnight to ensure complete dissolution of methyl cellulose^14^. Following this, egg yolk emulsion (Sigma-Aldrich, 17148) was added intermittently with constant stirring at room temperature to make a final solution containing 10% egg yolk emulsion. The viscosity of this 1X solution of mock sputum was measured using a cone and plate rheometer (MCR 301 Anton Paar, Graz, Austria). The reaction pads containing dried reagents were then rehydrated with 9 µl of different dilutions (1X, 2X and 5X) of mock sputum, each of which were spiked with 100 copies of *Mtb* genomic DNA. The compatibility of DNA amplification in FLIPP-NAAT was also tested with conventional methods of DNA extraction using methods described under Supplementary Methods.

### Testing of clinical samples in FLIPP-NAAT

A set of 30 clinical sputum samples obtained at Christian Medical College (CMC), Vellore, India were tested using sputum smear microscopy, Xpert MTB/RIF, and FLIPP-NAAT after all methods were approved by the CMC Institutional Review Board Ethics Committee. All standard operating procedures (SOPs) were in accordance with the RNTCP (Revised national tuberculosis control program) and C&DST (culture and drug susceptibility testing) guidelines. The set of clinical samples was diverse to include samples that were smear microscopy negative, smear microscopy positive and positive for other respiratory diseases but not TB. The Xpert MTB/RIF negative samples were culture positive at CMC Vellore for other microorganisms such as Viridans group of *Streptococci*, *Pseudomonas*, *Klebsiella*, *Candida tropicalis*, and *Candida glabrata*. Sputum smear microscopy and Cepheid Xpert MTB/RIF tests were conducted at CMC as part of routine clinical diagnostic procedures. DNA was extracted from the samples at CMC using a QIAamp DNA mini kit (Qiagen, 51304). 200 µl of native sputum sample was added to a Qiagen column and the DNA was extracted in a final volume of 100 µl. Extracted and purified DNA from the 30 clinical samples was then tested in FLIPP-NAAT in a blind study at IISc Bangalore, where only the sample identity numbers were known. The reaction was set-up as explained in the section on ‘DNA amplification in tube’, with two modifications.

First, the total volume of the reaction was reduced to 11.5 µl from 12.5 µl to ensure that the paper pads were not filled to capacity to completely prevent leakage issues (if any). Second, 3.5 µl of template DNA was added instead of 1 µl, eliminating additional water that was being added to fill the remaining volume. 3.5 µl of 100copies/µl solution of purified *Mtb* gDNA or water was added for positive and negative controls, respectively. After addition of the reaction mix into paper pads, the protocols followed for conducting the reaction and fluorescence imaging were as explained earlier. The following rules were used for interpreting test results: (i) the threshold intensity was set to be µ_N_ + 3σ_N_, where µ_N_ is the mean intensity from the three negative controls present in each device, and σ_N_ is the standard deviation of mean intensities across these negative controls, (ii) the test would be considered valid only if all the three positive controls in the device showed amplification (mean intensity >100) while none of the three negative controls showed amplification (mean intensity <100), (iii) Any sample with intensity greater than the threshold was considered as a positive while a sample with mean intensity below the threshold was considered as a negative, (iv) Each sample was tested in duplicates and a positive test result for at least one replicate was interpreted as a TB-positive test, (v) To address the challenges faced while testing of smear-negative samples, each smear negative sample was tested in six replicates and a positive test result for at least one replicate was interpreted as a TB-positive test.

## Results

### FLIPP NAAT: Design, fabrication, and user experience

**Figure 1:**
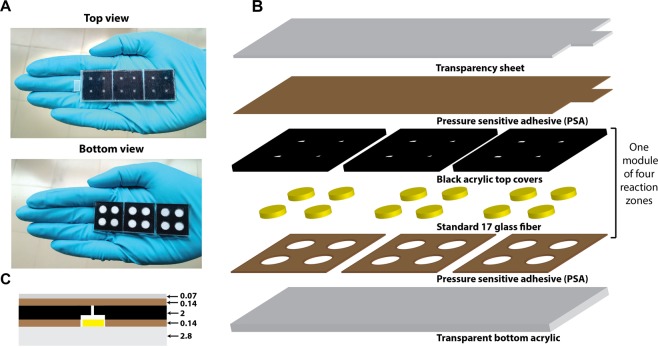
FLIPP-NAAT design. (**A**) Top and bottom view of the fabricated device (**B**) Schematic of the layer-by-layer assembly of the device. (**C**) Schematic of front view of device demonstrating the position of paper (yellow) between the bottom acrylic (grey) and the engraved top acrylic (black). The color of the materials has been kept same as in (**B**) and thickness of each layer is marked in millimeters.

The top and bottom view of a 12-reaction-zone FLIPP NAAT device is shown in Fig. 1A and a schematic of the layer-by-layer assembly is shown in Fig. 1B. The device consists of a 7.7 cm × 2.6 cm rectangular base constructed from 2.8 mm thick transparent acrylic. Three 2.5 cm × 2.5 cm PSA sheets, each having four equidistant circular holes (6.75 mm diameter), were adhered on the base with a gap of 1 mm between them. Each 2.5 cm × 2.5 cm PSA represented one section/module of the device (Fig. 1B). In each module, four circular reaction pads (6.3 mm diameter) made from Standard 17 were placed within the 6.75 mm circular boundaries defined by the PSA sheets, such that the pads directly contacted the acrylic base. The top covers for each section were constructed from 2 mm thick black acrylic. For each module, a 2.5 cm × 2.5 cm acrylic cover comprised of four circles (6.75 mm diameter), engraved to a depth of ~600 um, such that they aligned with the holes in the PSA (Fig. 1C). The engraved regions provided tightly closed reaction zones for each reaction pad. For addition of reagents, 2 mm × 2 mm square openings were cut in the top cover at the center of each reaction zone. This assembly was pressurized using a vice to ensure that the bottom and top acrylic sections stick well to each other. The top seal of the device consisted of a combination of PSA and a transparency sheet cut to match the dimensions of the acrylic base, with an additional flap to ease peeling off. After addition of reagents, the top seal was pressed hard manually to ensure a good seal.

**Figure 2:**
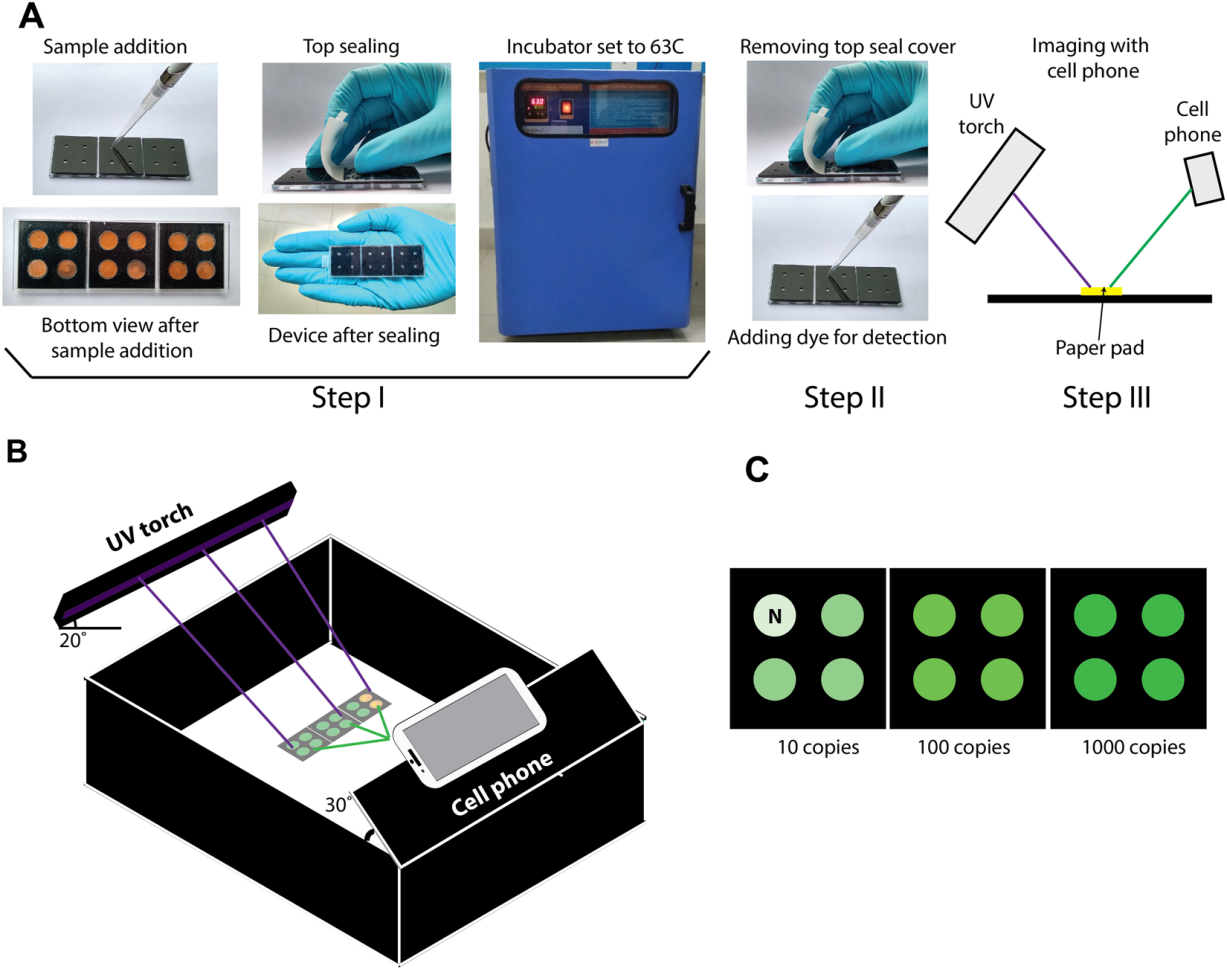
User experience of conducting a NAAT in FLIPP-NAAT. (**A**) User steps involved in operating FLIPP-NAAT. Step I: Sample addition to FLIPP-NAAT, sealing the device and placing it in an incubator set to the reaction temperature. Step II: Removing FLIPP-NAAT from the incubator and adding the DNA intercalating dye. Step III: A UV torch is used for excitation and results are captured using a cell phone camera. (**B**) Schematic of an inexpensive imaging set-up for end-point fluorescence detection. (**C**) Schematic of expected TB test results from FLIPP-NAAT. In one implementation, green fluorescence intensity increases with increasing number of target molecules.

Conducting a NAAT in FLIPP-NAAT involves a simple three-step user experience (Fig. 2A). The first step involves addition of the samples to the device, putting the top seal, and placing the device in an incubator (Step I; Fig. 2A). The second step involves removing the device from the incubator and adding a DNA-intercalating fluorescent dye (Step II; Fig. 2A). The last step involves capturing an image of test results using a cell phone camera (Step III; Fig. 2A). Figure 2B shows the schematic of the imaging box built in-house, demonstrating positions of the UV torch and the cell phone. A schematic of expected results shows green fluorescence in the presence of 10, 100, and 1000 copies of target DNA (Fig. 2C) and lack of green fluorescence in the absence of target (N; Fig. 2C). In one implementation, each module can be used for testing an independent sample in duplicates along with a positive and a negative control and hence 3 samples can be tested on the device at a time. In an alternate implementation, a 3-module device can be used for conducting as many as 6 replicates of a single sample along with 3 positive and 3 negative controls, to increase the probability of detection in samples with low bacillary load.

### Analytical sensitivity and specificity of the LAMP assay

**Figure 3:**
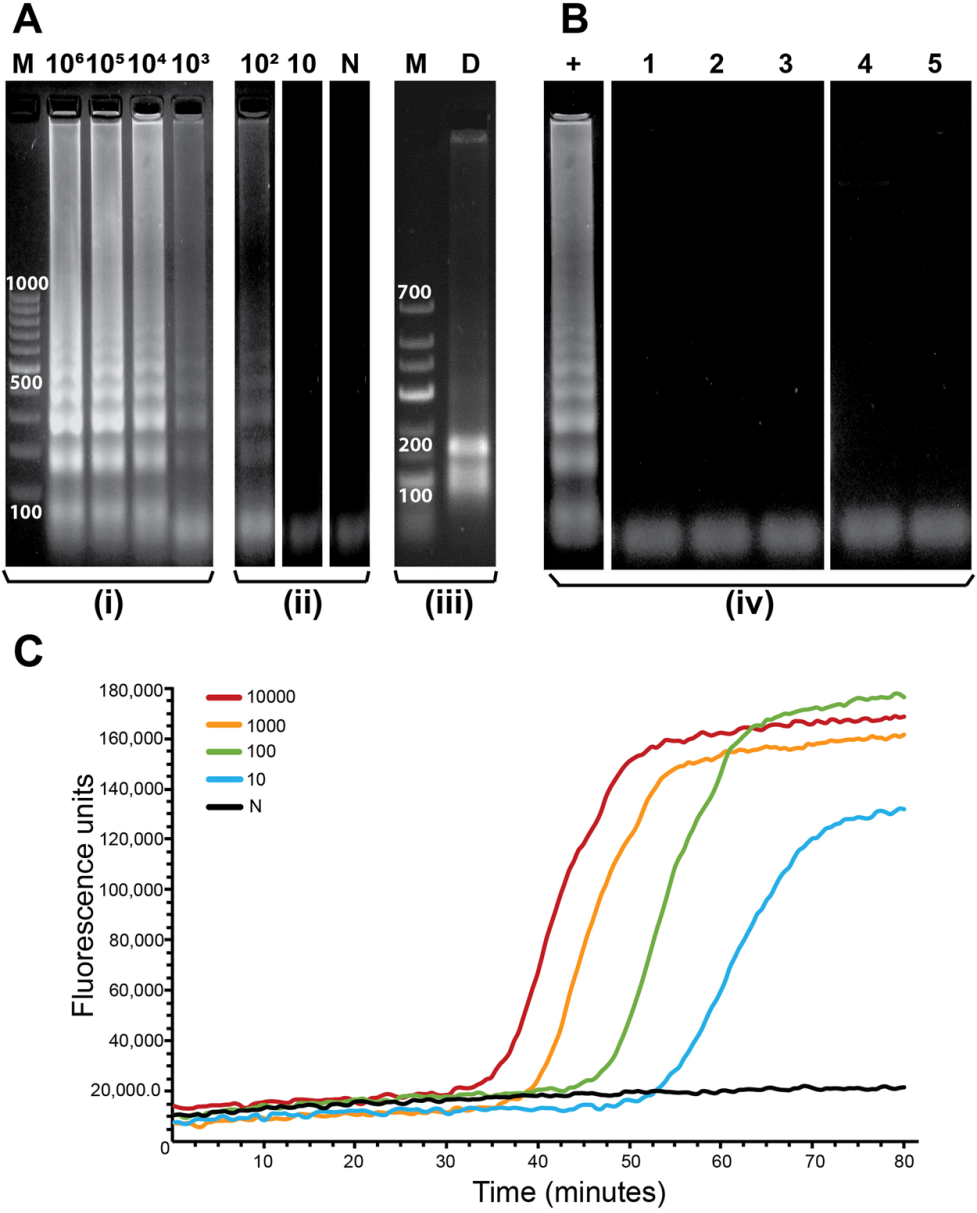
LAMP in tube. (**A**) Gel electrophoresis analysis of products of LAMP reactions conducted in tube with varying starting copy numbers of *Mtb* gDNA (10^6^ copies up to 10 copies). The numbers on top of the lanes indicate the starting copy number of the template. N- No template control, M- DNA marker and D- digested LAMP amplicons. Reaction time was 40 minutes. (**B**) Specificity of the LAMP assay tested against non-*Mtb* targets. +: Positive control (*Mtb* gDNA), 1: Human gDNA, 2: *E. coli* gDNA, 3: Mycobacterium smegmatis gDNA, 4: plasmid with hepatitis C insert, 5: plasmid with dengue insert. (**C**) Real-time amplification curves for LAMP in tube. Reaction time was 80 minutes. (i–iv) denote lanes from different gels. Full-length gels are presented in Supplementary Fig. S9 (i), (ii), (iii) and (iv).

The limit of detection (LOD) of the LAMP assay in tube, using gel electrophoresis as the detection method, was determined to be 100 starting copies of *Mtb* genomic DNA for a 40-minute reaction. Figure 3A shows the results of gel electrophoresis conducted on products of LAMP reactions starting from 10^6^ copies, serially diluted 10x, down to 10 copies. A ladder-like pattern of amplified DNA products, typical of LAMP reactions, was observed for reactions starting from 10^6^ to 10^2^ copies, but not for 10 copies. The negative control (lane N; Fig. 3A) showed no amplification. Digestion of LAMP amplicons was theoretically expected to produce bands of size 100 and 160 bp, which were observed on the gel (lane D; Fig. 3A), confirming target-specific amplification. The analytical specificity of the reaction was tested against five different non-*Mtb* targets and no amplification was observed for any of these (Fig. 3B). Additionally, Bi *et al*.^13^ had tested the analytical specificity of this assay against 22 species of non-tuberculous mycobacteria, 7 non-mycobacterial species that present *Mtb*-like symptoms and 50 *Mtb* clinical isolates. This was a crucial factor for selection of this assay for use in FLIPP-NAAT. The LAMP assay was also found to be tolerant to high concentrations of human gDNA for a reaction time of 80 minutes. Target-specific amplification was observed for the positive controls even in the presence of varying concentrations of human gDNA while the negative controls containing only human gDNA showed no amplification (see Supplementary Fig. S1).

### Real-time LAMP reactions

Out of the four DNA-intercalating dyes tested, only SYBR Safe worked with this LAMP assay for real-time detection and the concentration of the dye in the LAMP mix was optimized to be 3X (where 1X is the manufacturer-recommended concentration for use in agarose gels). At concentrations below 3X, we observed that the signal to noise ratio was very low and concentrations above 3X adversely affected the reaction kinetics, delaying the lift-off times. Real-time curves using the optimum dye concentration revealed that the limit of detection of the assay could be improved by increasing reaction time. Real-time amplification curves for 10, 10^2^, 10^3^, and 10^4^ copies lifted off at approximately 52, 44, 38, and 34 minutes, respectively (Fig. 3C). At 80 minutes, real-time curves for all starting copy numbers saturated (Fig. 3C). For all further experiments, the reaction time was accordingly increased to 80 minutes to enable low copy number detection. The LOD in tube improved to 10 copies of *Mtb* gDNA with the 80-minute reaction.

### Porting the LAMP assay to paper

**Figure 4:**
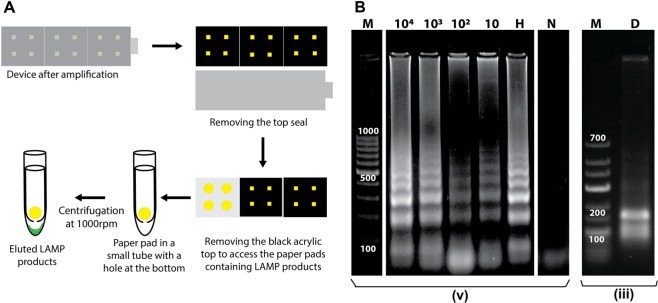
LAMP amplification in paper. (**A**) Schematic of the method used for retrieving the LAMP amplicons from the paper pads. (**B**) Gel electrophoresis analysis of products of LAMP reactions conducted in paper starting from varying number of starting *Mtb* gDNA copies (10^4^ up to 10). The numbers on top of the wells indicate the starting copy number of the template. H- 1000 copies of human gDNA were added to the reaction mix along with 1000 copies of *Mtb* gDNA; N- No template control; M- DNA marker; and D- digested LAMP amplicons. The reaction time was 40 minutes. Larger spaces between sections of lanes in a gel image indicate lanes from different gels and smaller spaces represent lanes from the same gel. Full-length gels are presented in Supplementary Fig. S9 (iii) and (v).

Before porting the assay into paper, its sensitivity to temperature was tested and the assay was found to be very sensitive to temperature. Amplification was observed only at temperatures of 61, 62 and 63 °C (see Supplementary Fig. S2), with maximum efficiency at 63 °C, which was chosen as the operating temperature. For the initial trials of porting the reaction to paper, amplicons were analyzed using gel electrophoresis to confirm that the amplification was target specific. Figure 4A demonstrates the method used for extracting the LAMP amplicons from the paper pads after the LAMP reaction. Figure 4B shows the gel results for LOD of 10 starting copies of *Mtb* gDNA in paper and the digestion of the amplicons confirmed target-specific amplification (D; Fig. 4B). Since sputum samples also carry human gDNA, the tolerance of the assay to foreign DNA was tested by adding 1000 copies of (15 ng) human gDNA along with 1000 copies of *Mtb* gDNA to the reaction. Target-specific amplification was still achieved (H; Fig. 4B) with no interference observed due to presence of human genomic DNA.

### DNA amplification and fluorescence detection in FLIPP-NAAT

Based on real-time LAMP curves, an 80 minute-reaction time was chosen for FLIPP-NAAT to enable detection at low copy numbers. Because fluorescence imaging is a crucial part of testing in FLIPP-NAAT, an economical imaging set-up (Fig. 2B) was designed and fabricated out of black acrylic to avoid any background fluorescence. The angle of the excitation source (20°) was optimized to maximize sample excitation and minimize reflection of the UV light from the device surface, which would otherwise be captured by the camera leading to background noise. The angle of the phone camera (30 ) was optimized to minimize capturing reflected UV light from the device surface. Out of the two fluorescent dyes tested, SYBR Green I was chosen for end-point detection. Though SYBR Safe was more specific to double stranded DNA and generated very little background for negative controls, its excitation peak lay in the UV range and it was very difficult to find an inexpensive material transmissive to UV. SYBR Green I interacts non-specifically with single stranded DNA which led to some background fluorescence in negative controls due to the presence of high concentration of LAMP primers. But because target-specific LAMP reactions produced large amounts of amplicons, the positives were distinguishable from the negatives using SYBR Green I.

**Figure 5:**
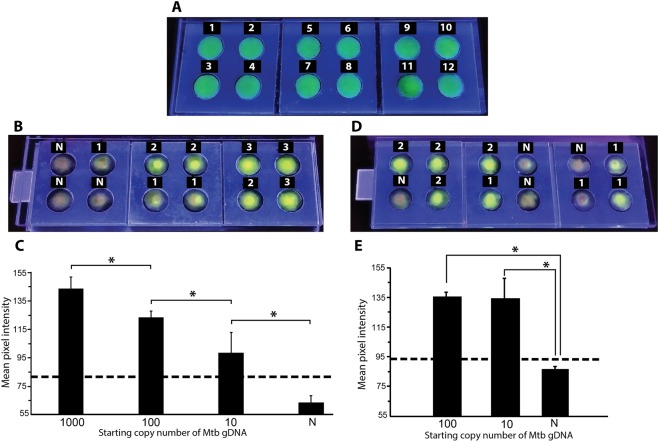
LAMP in FLIPP-NAAT and end-point fluorescence detection. (**A**) Testing uniformity of field in FLIPP-NAAT. Numbers on each reaction zone mark the 12 replicates. The mean intensity for signals from the 12 reaction zones was 160 with a coefficient of variation of only 5.4%. (**B**) Cell phone image of FLIPP-NAAT after 5 minutes of addition of SYBR Green I dye for fluorescence detection. Numbers on top of each reaction zone indicate log_10_ of *Mtb* gDNA starting copy number. N – no template control. (**C**) Mean pixel intensity of green color for the different starting copy numbers. The mean intensity of each starting copy number was statistically different from that of others (*P < 0.05; N = 3). (**D**) LAMP reactions in FLIPP-NAAT in presence of 3.1 * 10^3^ copies of human gDNA. Numbers on top of each reaction zone indicate log_10_ of *Mtb* gDNA starting copy number. N – no template control containing only human gDNA. (**E**) Mean pixel intensity of green color for the different starting copy numbers. The mean intensity of 100 and 10 copy number was statistically different from that of the negative controls (*P < 0.05; N = 3). The one drop out for 10 copies (second reaction zone from the right in the bottom row, Panel D) was not included for the intensity analysis as it was a false negative. The dashed line represents the threshold value of (µ_N_ + 3σ_N_). Samples with intensity below the threshold were considered as TB negative while samples with green color intensity above the threshold were considered as TB positive.

The uniformity of field for fluorescence imaging in FLIPP-NAAT was first tested (Fig. 5A) to ensure that the fluorescence was independent of the reaction zone’s location in the device. The mean green fluorescence intensity across 12 reaction zones had a standard deviation of ±9 intensity values and coefficient of variation of 5.4% (Fig. 5A). Hence, we did not find any significant difference in the mean intensities across locations. TB testing was then performed in FLIPP-NAAT with varying starting copy numbers of *Mtb* gDNA. The NTCs appeared slightly orange (N; Fig. 5B) as SYBR green I is orange in its native state while pads containing target DNA appeared green due to the DNA-dye complex formation (Fig. 5B). The intensity of green fluorescence increased with increasing starting copy numbers (Fig. 5B). The mean intensity for reaction zones containing 1000 starting copies of *Mtb* gDNA was statistically higher than 100 copies, for 100 copies was statistically higher than 10 copies, and for 10 copies was statistically higher than negative controls that did not contain *Mtb* gDNA (P < 0.05; N = 3; Fig. 5C). The threshold for a positive test (µ_N_ + 3σ_N_) is represented by the dashed line (Fig. 5C). The mean intensity for all samples containing *Mtb* gDNA was greater than the threshold value, enabling successful distinction from samples not containing *Mtb* gDNA. The tolerance to presence of human gDNA was also tested in FLIPP-NAAT. All the negative controls containing only human gDNA showed no amplification while samples containing both *Mtb* gDNA and human gDNA showed amplification (Fig. 5D,E). One dropout was observed for reactions set-up with 10 starting copies of *Mtb* gDNA. A green band pass filter (510–550 nm) was tested for reducing background fluorescence but was found to produce no significant improvement in the ability to distinguish between a positive and a negative test (see Supplementary Fig. S3).

### Performance of FLIPP-NAAT containing dried LAMP reagents

**Figure 6:**
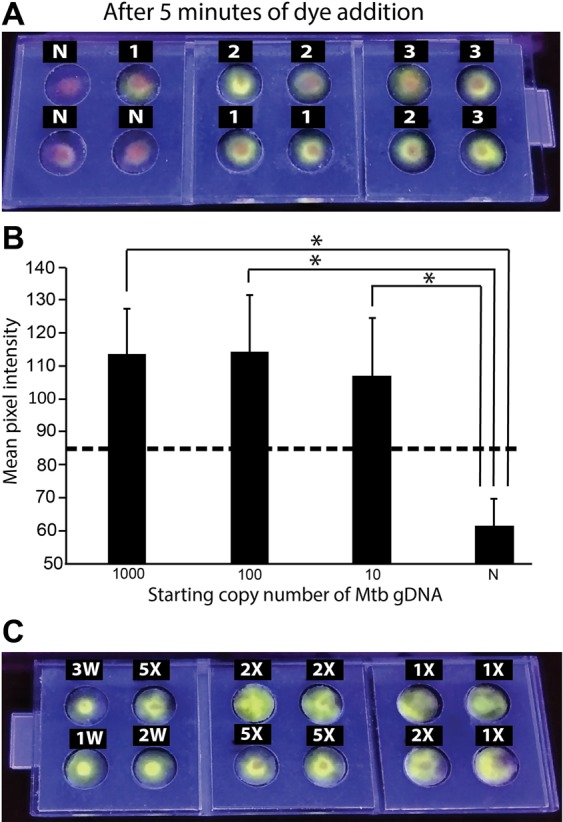
LAMP in FLIPP-NAAT from dry-stored reagents. (**A**) End-point imaging of the FLIPP-NAAT device after 5 minutes of addition of SYBR green I dye. Numbers on top of each reaction zone indicate log_10_ of starting copy number. N – no template control. (**B**) Mean pixel intensity of the green color from all starting copy numbers was statistically higher (*P < 0.05; N = 3) from that of no template control. The dashed line represents the threshold value of (µ_N_ + 3σ_N_). Samples with intensity below the threshold were considered as TB negative while samples with green color intensity above the threshold were considered as TB positive. (**C**) LAMP in FLIPP-NAAT from dry-stored reagents and rehydration with mock sputum. Each reaction zone had 100 copies of *Mtb* gDNA as the starting template. The markings on top of each of the reaction zone indicate the dilution of mock sputum used for rehydration. 3 W, 2 W, 1 W – reaction set-up with template suspended in water containing 1000, 100 and 10 copies of *Mtb* gDNA respectively, as positive controls. All reactions worked despite the presence of the complex sputum matrix.

LAMP reaction mixes were dry-stored in reaction pads within FLIPP-NAAT. Because trehalose is a well-known enzyme cryopreserving agent, a range of trehalose concentrations up to a maximum of 10% w/v were first tested for compatibility with the assay. No significant loss in amplification was observed at any concentration (see Supplementary Fig. S4). Ten per cent trehalose was therefore selected for dry-storage of LAMP reagents. Although the reaction volume for fresh reagents was set to 12.5 µl, we found that 12.5 µl was excessive for rehydration of paper pads containing lyophilized reagents, potentially because of residual glycerol in the lyophilized pads. In addition, when rehydrated with 12.5 µl, there was poor mixing of the amplicon solution with the fluorescent dye (added after completion of the reaction) because the pads were filled beyond capacity. Reducing the rehydration volume to 9 µl resolved this issue and allowed better mixing of the dye with amplicons. Bright green color was observed in reaction zones with 1000, 100 and 10 *Mtb* gDNA starting copies (3, 2 and 1 respectively, Fig. 6A) while the negative controls appeared predominantly orange (N; Fig. 6A). The mean fluorescence intensity for different copy numbers for positives were significantly distinguishable from the negative controls (P < 0.05; N = 3; Fig. 6B) and all the positives were above the threshold value (as indicated by the dashed line, Fig. 6B).

### Detection of TB from complex samples

Since sputum is a viscous fluid, it is important that DNA amplification techniques used in TB-NAATs be compliant with viscous sputum. To test robustness in the presence of sputum, FLIPP-NAAT test zones with dried reagents were rehydrated with different dilutions (1X, 2X and 5X) of mock sputum spiked with 100 copies of *Mtb* gDNA. The viscosity of 1X mock sputum was measured to be 350 cP. FLIPP NAAT successfully amplified DNA within undiluted (1X) and diluted (2X and 5X) mock sputum (Fig. 6C). Three positive controls with 1000, 100 and 10 starting copies of *Mtb* gDNA in water (3 W, 2 W and 1 W, respectively; Fig. 6C) were run on the same device. No significant reduction was observed in the intensity of green color from zones rehydrated with mock sputum as compared to zones rehydrated with water. As the next step, the compatibility of FLIPP NAAT with commonly used bacterial lysis and DNA extraction methods was tested; successful amplification was observed from DNA extracted using both a commercially available Qiagen kit and a crude culture boiling and centrifugation method (see Supplementary Methods and Supplementary Fig. S5).

### Testing of clinical samples in FLIPP-NAAT

**Figure 7:**
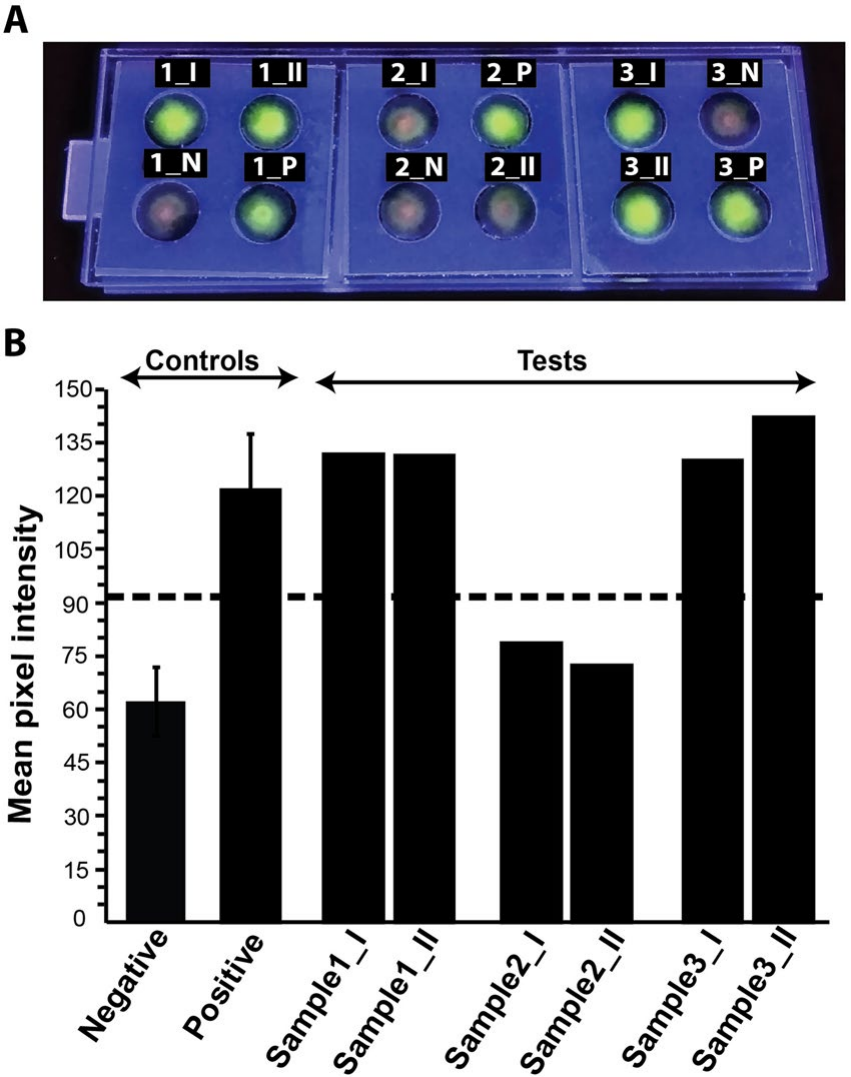
TB testing for clinical samples in FLIPP-NAAT. (**A**) Three clinical samples were tested per device with a negative and positive control in each module. Each sample was run in duplicates. Numbers on top of each zone represent the sample number and suffix ‘I’ and ‘II’ represent the duplicates. N – negative control and P – positive control. (**B**) Mean pixel intensity of green color from all the six blind clinical samples, negative and positive controls. The dashed line represents the threshold value of (µ_N_ + 3σ_N_). Samples with intensity below the threshold were considered as TB negative while samples with green color intensity above the threshold were considered as TB positive.

The results for TB testing of 30 clinical samples in a blind study in FLIPP-NAAT were interpreted according to the rules described in the materials and methods section. A representative result of testing three samples (in duplicates) in FLIPP-NAAT is shown in Fig. 7A. The validity of the test was confirmed by no amplification for negative controls (1_N, 2_N and 3_N; Fig. 7A) and amplification for positive controls (1_P, 2_P and 3_P; Fig. 7A). The emergence of bright green color spots for both the duplicates of Samples 1 and 3 (1_I, 1_II and 3_I, 3_II; Fig. 7A) indicated a positive test while absence of the bright green spot for both duplicates of Sample 2 (2_I and 2_II; Fig. 7A) indicated a negative test. These results were interpreted by image analysis (Fig. 7B) where the green color intensity for sample 1 and sample 3 was above the threshold (µ_N_ + 3σ_N_) while that for sample 2 was below the threshold. The end-point images for all the devices used for testing of clinical samples were analyzed in the same fashion (see Supplementary Figs S6–S8 and Table S2). When each sample was tested in duplicates, 21 samples reported TB positive and 9 samples reported TB negative with FLIPP-NAAT. As it was a blind study, Xpert MTB/RIF assay results were received after FLIPP-NAAT testing and were found to have 19 true positives and 11 true negatives. When compared to Xpert MTB/RIF assay, FLIPP-NAAT reported 5 false positives (Samples 11,20, 27, 28 and 30) and 3 false negatives (Samples 21, 24 and 25) resulting in a clinical sensitivity of 86.36% and clinical specificity of 68.75%. It was further observed that the clinical sensitivity of FLIPP-NAAT was 100% for smear-positive samples but dropped to 86.36% when smear-negative samples were included in the analysis. This loss in clinical sensitivity was addressed by retesting the smear-negative TB-positive samples by conducting six replicates of each sample (instead of duplicates) in a 3-module FLIPP NAAT device. Incorporation of more replicates increased the clinical sensitivity for the entire set of 30 samples to 100% while the clinical specificity remained 68.75%. To investigate the imperfect specificity, the cases of false positives were analyzed further. Amplicons from these FLIPP NAAT test zones were loaded on agarose gels, before and after restriction enzyme digestion (see Supplementary Figs S7 and S8A,B). The gel patterns for the false positive samples were very different from true positives (see Supplementary Fig. S8A) and these samples did not undergo enzyme digestion (see Supplementary Fig. S8B), confirming that the amplification was not specific to *Mtb*. The final positive and negative predictive values for the set of 30 clinical samples was 79.16 and 100%, respectively. FLIPP-NAAT, smear microscopy, and Xpert MTB/RIF results for the 30 clinical samples have been compiled in Table 1.

**Table 1:** Comparison of FLIPP-NAAT with smear microscopy and Xpert MTB/RIF.

Study No.	Smear	Xpert MTB/RIF assay	VL/L/M/H	FLIPP-NAAT
1	3+	Mtb+	H	Mtb+
2	No AFB	Negative	N/A	Negative
3	2+	Mtb+	H	Mtb+
4	3+	Mtb+	H	Mtb+
5	No AFB	Negative	N/A	Negative
6	No AFB	Negative	N/A	Negative
7	2+/3+	Mtb+	H	Mtb+
8	3+	Mtb+	M	Mtb+
9	Sc	Mtb+	M	Mtb+
10	2+	Mtb+	L	Mtb+
11	No AFB	Negative	N/A	Mtb+
12	2+	Mtb+	M	Mtb+
13	1+	Mtb+	M	Mtb+
14	3+	Mtb+	M	Mtb+
15	No AFB	Negative	N/A	Negative
16	2+	Mtb+	L	Mtb+
17	1+	Mtb+	L	Mtb+
18	3+	Mtb+	M	Mtb+
19	1+	Mtb+	M	Mtb+
20	No AFB	Negative	N/A	Mtb+
21	No AFB	Mtb+	L	Mtb+
22	No AFB	Mtb+	VL	Mtb+
23	No AFB	Mtb+	L	Mtb+
24	No AFB	Mtb+	VL	Mtb+
25	No AFB	Mtb+	VL	Mtb+
26	No AFB	Negative	N/A	Negative
27	No AFB	Negative	N/A	Mtb+
28	No AFB	Negative	N/A	Mtb+
29	No AFB	Negative	N/A	Negative
30	No AFB	Negative	N/A	Mtb+

VL: Very low, L: Low, M: Medium, H: High.

## Discussion

Despite some challenges that surfaced during testing of clinical samples, the results of FLIPP-NAAT testing are encouraging. The following sections elaborate upon the issues related to clinical sensitivity and specificity.

### Clinical sensitivity

When all samples were tested in duplicates, the sensitivity for smear-positive samples was 100%, but the pooled sensitivity including smear-negative samples was 86.36%. This loss is attributed to the low bacterial load present in smear-negative samples leading to stochasticity in the presence of target DNA in the 3.5 µl of sample added to the device. This limitation can be addressed in two ways. First, sample preparation could be improved to suit the needs of FLIPP NAAT. The generic Qiagen extraction kit used in this work recommended that DNA be extracted in 100 µl final volume, only a small fraction of which could be tested in a single FLIPP-NAAT test zone. Alternate sample preparation techniques that elute DNA in smaller volumes will improve the clinical sensitivity of FLIPP NAAT. Nevertheless, resetting the clinical sensitivity back to 100% by increasing the number of test replicates demonstrates the ability of FLIPP-NAAT to detect TB from a wide range of samples having varying loads of *Mtb* (as indicated in Table 1). The second method of potentially enhancing sensitivity is to conduct FLIPP NAAT testing from dried reagents, which will enable using up to 9 µl of sample per test zone, ~2.5x higher than the volume accommodated in the current tests. Preliminary results of successful amplification from dried reagents are presented in this work. Even though the above-mentioned challenges exist, the current performance of FLIPP-NAAT with clinical samples substantiates its potential to be a higher-sensitivity replacement for smear microscopy.

### Clinical specificity

The clinical specificity of FLIPP-NAAT could be improved further. Loss in specificity ensued from the use of a sequence-independent DNA intercalating fluorescent dye. The finding that all false positive reactions generated *Mtb*-independent amplicons reveals a path to recover the lost specificity by introducing sequence-specific detection probes in FLIPP-NAAT, which will be the topic of our future work.

Conventionally, NAATs have been conducted in tubes placed inside a thermal cycler. Tubes have an associated form factor and require specially designed heating blocks and instruments. A heated and heavy top cover is also required to prevent the tubes from opening due to generation of vapors at high temperature and to ensure that the vapors do not condense. Lack of proper sealing could lead to carry-over contamination which is highly undesirable for diagnostic devices. Fluorescence imaging in tubes is challenging – multiple internal reflections make it difficult to differentiate positives from negatives and to provide quantitative results. Paper, on the other hand, is flat and does not require any particular form factor-accommodating infrastructure. Fluorescence imaging is more reliable as shown in the results for fluorescence imaging in FLIPP-NAAT. Compared to tubes, FLIPP-NAAT also presents a mixing advantage. Conducting NAATs from dry stored reagents in tubes requires vortexing to mix the rehydrated reagents followed by centrifuging to push the contents to the bottom of the tube. On the other hand, FLIPP-NAAT is designed such that mixing is automatic on rehydration, reducing the requirement of ancillary equipment, making it more point-of-care compatible. While several isothermal nucleic acid amplification techniques exist, we chose LAMP for FLIPP-NAAT because of its multiple distinctive characteristics (see Supplementary Note S1).

There have recently been three reports on paper-based NAAT devices, designs of which appear similar to the eye as that of FLIPP NAAT^15–17^. However, the fabrication techniques, working principles, flexibility in device design, imaging set-up and costs involved in making FLIPP NAAT are strikingly different (see Supplementary Note S2). One of the most desirable features of FLIPP NAAT is that the material cost of making a 12-reaction-zone device is only $0.88 and the cost of reagents required per reaction zone is $0.43 (see Supplementary Tables S3 and S4). Modular design is a powerful feature of FLIPP-NAAT and using the current definition of a module, devices may be designed to contain 4 N reaction zones (where N = 1, 2, 3…), without any major modification in fabrication methods. The device design has been optimized (see Supplementary Note S3) as a simple layer-by-layer assembly of paper pads, plastics, and adhesives, and therefore it is compatible with mass manufacturing using injection molded plastic components.

Although this report focuses on TB diagnosis, FLIPP-NAAT is a platform technology for conducting NAATs by minimally trained users. FLIPP-NAAT is amenable to the detection of any DNA or RNA target from any sample type, provided it is appended to an appropriate sample-preparation technique. Because paper can be used to conduct many types of isothermal amplification techniques^11^, FLIPP-NAAT is not restricted to LAMP and can accommodate other isothermal amplification chemistries. Given its semi-quantitative fluorescence detection capabilities, FLIPP-NAAT can potentially be used for detection of fold-changes in gene expression. While it is a tool designed to be compatible with limited resource settings, it will also find applications in healthcare settings like clinics, hospitals, and beyond healthcare, in classrooms and teaching laboratories. It is a tool to make molecular diagnostics accessible, fast, easy, and affordable for all.

## Supplementary information

Compiled Supplementary Information

## Data Availability

All data generated or analyzed during this study are included in this published article (and its Supplementary Information Files).
