# Supplementary material for: A modular paper-and-plastic device for tuberculosis nucleic acid amplification testing in limited-resource settings
Source: Sci Rep. 2019 Oct 25;9:15367. doi: 10.1038/s41598-019-51873-8 (PMC6814773; doi:10.1038/s41598-019-51873-8)
Supplement: Supplementary file 1 — Compiled Supplementary Information [file 41598_2019_51873_MOESM1_ESM.pdf]

# A modular paper-and-plastic device for tuberculosis nucleic acid amplification testing in limited-resource settings

Navjot Kaur[a], Joy S. Michael[b], Bhushan J. Toley[a]*

[a] Department of Chemical Engineering
Indian Institute of Science
Bangalore, India
560012

[b] Department of Microbiology
Christian Medical College
Vellore, India
632004

*Correspondence to:
Bhushan J. Toley
Department of Chemical Engineering
Indian Institute of Science
Bangalore, India, 560012
Phone: +91-80-22933114
Email: bhushan@iisc.ac.in

## Supplementary Information

**Supplementary Table S1**. Sequence of the primers used for loop-mediated isothermal amplification (LAMP) targeting the hspX gene of Mycobacterium tuberculosis

| Primer | Sequence | Length |
|---|---|---|
| hspXF3 | TCATTCGCCGGACTCCG | 17 |
| hspXB3 | GGAACCGTACGCGAATTCC | 19 |
| hspXFIP | ACCTCGTAGCGCCCCTCTTACCTTCGACACCCGGTTGA | 38 |
| hspXBIP | GGACGTCGACATTATGGTCCGCGTCGAAGTCCTTCTGCTCG | 41 |
| hspXFLP | CATCTCGTCTTCCAGCCGCA | 20 |
| hspXBLP | GATGGTCAGCTGACCATCAAGGC | 23 |

**Supplementary Table S2.** Intensity analysis for TB testing from clinical samples in FLIPP-NAAT

| FLIPP-NAAT devices in Figure S7 | | | | | | | |
|---|---|---|---|---|---|---|---|
| Panel A | | Panel B | | Panel C | | Panel D | |
| Sample | Mean Intensity | Sample | Mean Intensity | Sample | Mean Intensity | Sample | Mean Intensity |
| 1_I | 131.694 | 4_I | 148.338 | 7_I | 131.717 | 10_I | 140.269 |
| 1_II | 131.155 | 4_II | 132.139 | 7_II | 138.828 | 10_II | 148.547 |
| 1_N | 59.919 | 4_N | 75.363 | 7_N | 96.425 | 10_N | 88.165 |
| 1_P | 103.337 | 4_P | 133.205 | 7_P | 136.012 | 10_P | 144.787 |
| 2_I | 78.599 | 5_I | 80.15 | 8_I | 135.932 | 11_1 | 146.598 |
| 2_II | 72.357 | 5_II | 93.591 | 8_II | 139.167 | 11_2 | 140.289 |
| 2_N | 72.792 | 5_N | 86.866 | 8_N | 139.48 | 11_N | 81.309 |
| 2_P | 129.084 | 5_P | 144.155 | 8_P | 136.698 | 11_P | 121.68 |
| 3_I | 130.02 | 6_I | 74.723 | 9_I | 125.111 | 12_I | 136.363 |
| 3_II | 142.195 | 6_II | 83.169 | 9_II | 137.017 | 12_II | 125.492 |
| 3_N | 53.529 | 6_N | 80.517 | 9_N | 95.614 | 12_N | 87.555 |
| 3_P | 132.077 | 6_P | 143.867 | 9_P | 144.731 | 12_P | 124.047 |
| Average N, µ | 62.080 | | 80.915 | | **FAILED TEST** | | 85.676 |
| Stdev N, σ | 9.812 | | 5.762 | | | | 3.794 |
| **µ + 3σ** | **91.515** | | **98.201** | | | | **97.060** |
| | | | | | | | |

| **Panel E** | | **Panel F** | | **Panel G** | | **Panel H** | |
|---|---|---|---|---|---|---|---|
| Sample | Mean Intensity | Sample | Mean Intensity | Sample | Mean Intensity | Sample | Mean Intensity |
| 13_I | 148.164 | 16_I | 137.858 | 19_I | 107.401 | 7_I | 138.551 |
| 13_II | 152.811 | 16_II | 153.844 | 19_II | 103.921 | 7_II | 136.752 |
| 13_N | 74.106 | 16_N | 79.293 | 19_N | 72.018 | 7_N | 62.42 |
| 13_P | 128.795 | 16_P | 145.859 | 19_P | 144.443 | 7_P | 130.96 |
| 14_I | 144.289 | 17_I | 98.983 | 20_I | 141.318 | 9_I | 77.025 |
| 14_II | 136.75 | 17_II | 127.779 | 20_II | 140.77 | 9_II | 72.347 |
| 14_N | 73.539 | 17_N | 94.537 | 20_N | 75.266 | 9_N | 68.308 |
| 14_P | 142.134 | 17_P | 135.309 | 20_P | 155.756 | 9_P | 140.805 |
| 15_I | 97.84 | 18_I | 125.964 | 8_I | 154.339 | 17_I | 62.362 |
| 15_II | 79.662 | 18_II | 122.638 | 8_II | 151.342 | 17_II | 104.034 |
| 15_N | 59.614 | 18_N | 68.203 | 8_N | 77.026 | 17_N | 70.985 |
| 15_P | 136.897 | 18_P | 122.987 | 8_P | 136.573 | 17_P | 119.705 |
| Average N, μ | 69.086 | | 80.678 | | 74.770 | | 67.238 |
| Stdev N, σ | 8.208 | | 13.221 | | 2.541 | | 4.382 |
| **μ + 3σ** | **93.711** | | **120.342** | | **82.392** | | **80.383** |

| **Panel I** | | **Panel J** | | **Panel K** | | **Panel L** | |
|---|---|---|---|---|---|---|---|
| Sample | Mean Intensity | Sample | Mean Intensity | Sample | Mean Intensity | Sample | Mean Intensity |
| 21_I | 93.876 | 24_I | 77.219 | 27_I | 90.755 | 30_I | **116.512** |
| 21_II | 85.786 | 24_II | 75.518 | 27_II | 133.282 | 30_II | **117.412** |
| 21_N | 90.362 | 24_N | 96.528 | 27_N | 89.745 | 30_N | **84.555** |
| 21_P | 165.242 | 24_P | 141.188 | 27_P | 163.996 | 30_P | **172.742** |
| 22_I | 93.154 | 25_I | 85.603 | 28_I | 151.157 | 23_I | **166.102** |
| 22_II | 137.185 | 25_II | 102.303 | 28_II | 135.874 | 23_II | **164.343** |
| 22_N | 81.847 | 25_N | 95.482 | 28_N | 78.497 | 23_N | **85.645** |
| 22_P | 175.221 | 25_P | 154.224 | 28_P | 186.651 | 23_P | **163.813** |
| 23_I | 100.309 | 26_I | 95.077 | 29_I | 87.29 | 27_I | **137.726** |
| 23_II | 108.33 | 26_II | 78.459 | 29_II | 78.448 | 27_II | **126.759** |
| 23_N | 84.875 | 26_N | 83.922 | 29_N | 70.855 | 27_N | **63.396** |
| 23_P | 140.001 | 26_P | 154.87 | 29_P | 141.929 | 27_P | **124.234** |
| Average N, μ | 85.683 | | 91.977 | | 79.699 | | **77.865** |
| Stdev N, σ | 4.297 | | 6.996 | | 9.502 | | **12.543** |
| **μ + 3σ** | 98.573 | | 112.964 | | 108.206 | | **115.493** |

| FLIPP-NAAT devices in Figure S8 | | | | | | | |
|---|---|---|---|---|---|---|---|
| **Panel A** | | **Panel B** | | **Panel C** | | **Panel D** | |
| Sample | Mean Intensity | Sample | Mean Intensity | Sample | Mean Intensity | Sample | Mean Intensity |
| 21_I | 135.925 | 22_I | 130.042 | 23_I | 132.657 | 24_I | **77.424** |
| 21_II | 147.213 | 22_II | 120.756 | 23_II | 101.311 | 24_II | **68.613** |
| 21_N | 89.659 | 22_N | 86.674 | 23_N | 86.364 | 24_N | **77.414** |
| 21_P | 133.738 | 22_P | 128.716 | 23_P | 147.642 | 24_P | **162.502** |
| 21_III | 146.462 | 22_III | 160.487 | 23_III | 117.306 | 24_III | **101.973** |
| 21_IV | 99.231 | 22_IV | 103.990 | 23_IV | 114.856 | 24_IV | **82.618** |
| 21_N | 89.728 | 22_N | 92.934 | 23_N | 84.712 | 24_N | **78.381** |
| 21_P | 153.242 | 22_P | 154.586 | 23_P | 163.857 | 24_P | **182.721** |
| 21_V | 135.248 | 22_V | 101.872 | 23_V | 133.351 | 24_V | **78.424** |
| 21_VI | 75.450 | 22_VI | 125.855 | 23_VI | 92.415 | 24_VI | **77.593** |
| 21_N | 99.034 | 22_N | 75.571 | 23_N | 95.106 | 24_N | **63.299** |
| 23_P | 127.270 | 22_P | 124.525 | 23_P | 156.42 | 24_P | **146.148** |
| Average N, μ | 92.807 | | 85.060 | | | | **73.031** |
| Stdev N, σ | 5.393 | | 8.793 | | | | **8.442** |
| **μ + 3σ** | 108.986 | | 111.440 | | | | **98.358** |
| | | | | | | | |
| **Panel E** | | | | | | | |
| Sample | Mean Intensity | Sample | Mean Intensity | Sample | Mean Intensity | Sample | Mean Intensity |
| 25_I | 153.558 | 25_II | 96.774 | 25_III | 127.915 | 25_IV | **101.607** |
| 25_V | 104.304 | 25_VI | 69.424 | 25_N | 79.628 | 25_P | **192.299** |
| 25_N | 98.735 | 25_P | 143.420 | 25_N | 78.534 | 25_P | **145.520** |
| Average N, μ | 85.632 | | | | | | |
| Stdev N, σ | 11.360 | | | | | | |
| **μ + 3σ** | 119.714 | | | | | | |

**Supplementary Table S3**. Cost of materials used for fabricating one device

| **Material** | **Size (cm)** | **Area, ($cm^2$)** | **Cost ($)** | **Cost/ cm2 ($)** | **Area per device ($cm^2$)** | **Cost ($)** |
|---|---|---|---|---|---|---|
| PSA sheet, A4 | 21x9.7 | 623.7 | 3.34 | 0.0053 | 38.92 | 0.624 |
| Transparency sheet, A4 | 21x29.7 | 623.7 | 0.04 | 0.00007 | 20.17 | 0.001 |

| | | | | | | |
|---|---|---|---|---|---|---|
| 2.8mm transparent acrylic sheet, A3 | 29.7x42 | 1247.4 | 5.61 | 0.0045 | 20.02 | 0.090 |
| 2mm black acrylic sheet, A3 | 29.7x42 | 1247.4 | 5.61 | 0.0045 | 18.75 | 0.084 |
| Standard 17 sheet, A4 | 21x29.7 | 623.7 | 14.25 | 0.0228 | 3.741 | 0.085 |
| Total material cost for one device ($) | | | | | | **0.885** |

**Supplementary Table S4**. Cost of reagents required for 1000 reactions

| **Reaction component** | **Price ($)** | **Stock conc and volume** | **Used per 12.5ul reaction** | **Cost/1000 reactions, ($)** |
|---|---|---|---|---|
| FIP | 25.652 | 150,100µM | 1.6µM, 0.25µl | 0.684 |
| BIP | 25.652 | 150,100µM | 1.6µM, 0.25µl | 0.684 |
| F3 | 18.526 | 350,100µM | 0.2µM, 0.25µl | 0.026 |
| B3 | 18.526 | 350,100µM | 0.2µM, 0.25µl | 0.026 |
| LF | 18.526 | 350,100µM | 1.2µM, 0.375µl | 0.318 |
| LB | 18.526 | 350,100µM | 1.2µM, 0.375µl | 0.318 |
| dNTPs | 83.59 | 10mM, 500µl | 1.25µl | 208.992 |
| DEPC Water | 8.693 | 100ml | 2.25µl | 0.196 |
| Pol, Buffer, MgSO4 | 103.235 | 8000U/ml, 1600 units | - | |
| Pol (70% cost) | 72.264 | 8000U/ml, 1600 units | 4unit | 180.66 |
| Buffer (20%cost) | 20.647 | 10X, 1500µl | 1X, 1.25µl | 1.721 |
| MgSO4 (10% cost) | 10.324 | 100mM, 1500µl | 6mM, 1µl | 0.413 |
| Betaine | 32.736 | 5M, 1500µl | 0.9M,2.25µl | 8.838 |
| SYBR green I | 539.290 | 10000X, 500µl | 50X,5µl | 26.965 |
| Total cost for **1000** reactions | | | | 429.843 |

**Supplementary Figure S1**. LAMP reactions in presence of different concentrations of human gDNA

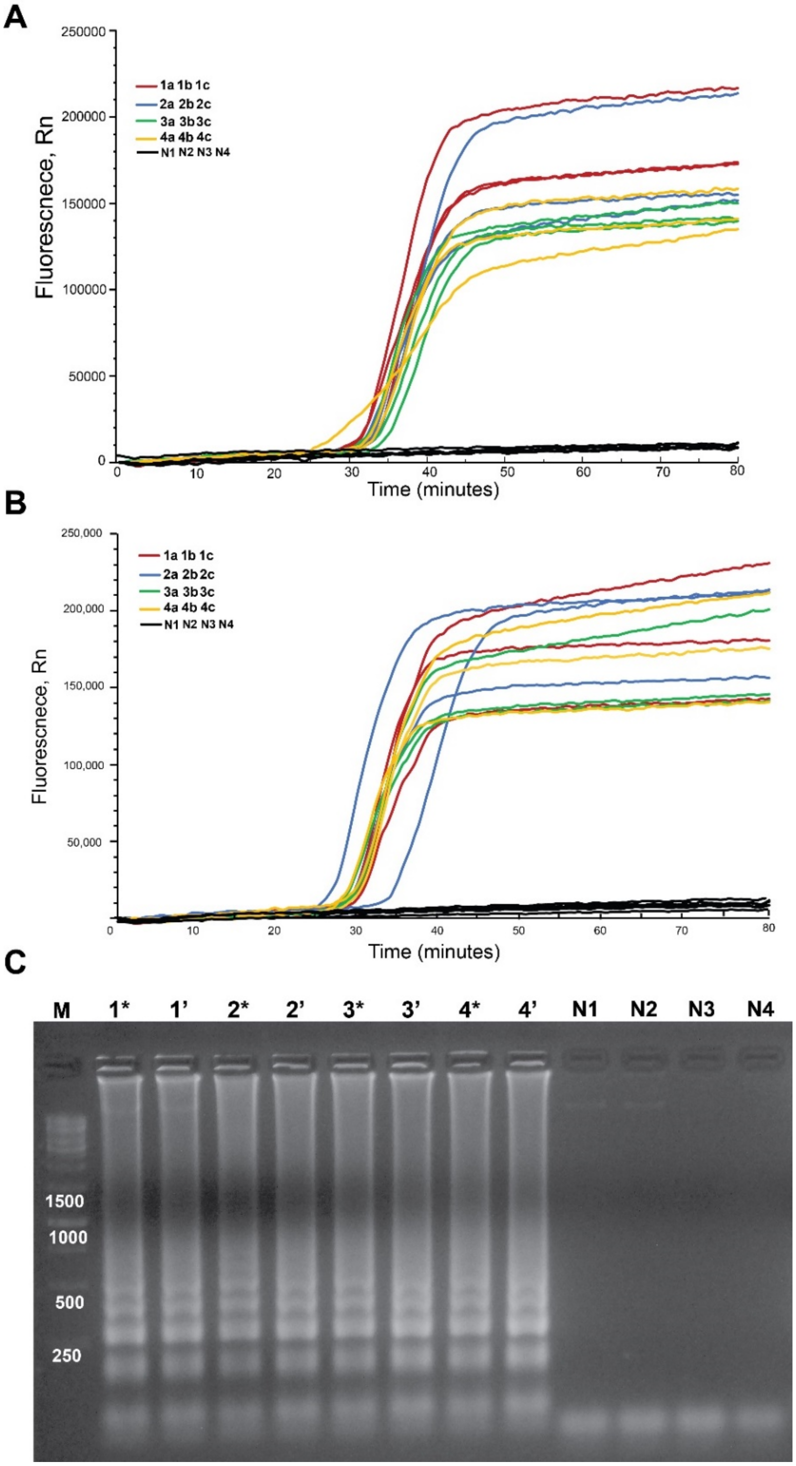


**Supplementary Figure S1**. LAMP reactions in presence of different concentrations of human gDNA for a reaction time of 80 minutes. Real-time amplification curves for LAMP reactions run with (A) 100 starting copies of Mtb gDNA and (B) 1000 starting copies of Mtb gDNA in presence of different

concentrations of human gDNA. (C) Gel electrophoresis analysis of the LAMP amplicons. 1- $3.1*10^4$, 2- $3.1*10^3$, 3- $3.1*10^2$, 4- 3.1*10 copies of human gDNA respectively. a, b and c represent triplicates. N- No template control was run in the presence of different concentrations of human gDNA as indicated by 1,2,3 and 4 but no Mtb gDNA was added. * - 100 starting Mtb gDNA copies and '– 1000 starting Mtb gDNA copies. All samples were run in triplicates. All the lanes are from the same gel and the original gel image has been used.

**Supplementary Figure S2**. Optimization of the reaction temperature

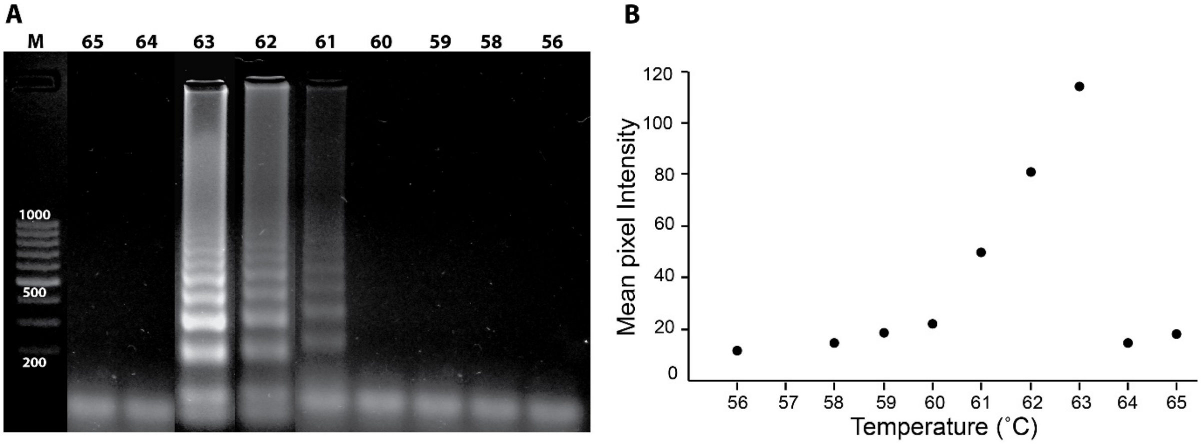


**Supplementary Figure S2**. Temperature optimization for the LAMP reaction. (A) Gel electrophoresis analysis of LAMP amplicons. Numbers on top of each well indicate the operating reaction temperature in ˚C. The reaction time was 40 minutes. (B) The average pixel intensity for each lane, calculated using ImageJ. Maximum amplification was observed at 63˚C.

A rectangular selection area covering each lane from the well till the bottom was drawn in ImageJ and the mean intensities were calculated. The mean pixel intensity, which is proportional to the total amount of DNA produced was plotted against the respective reaction temperature. Maximum amplification was observed at 63˚C, which was hence chosen as the reaction temperature.

**Supplementary Figure S3**. Fluorescence imaging using optical filter

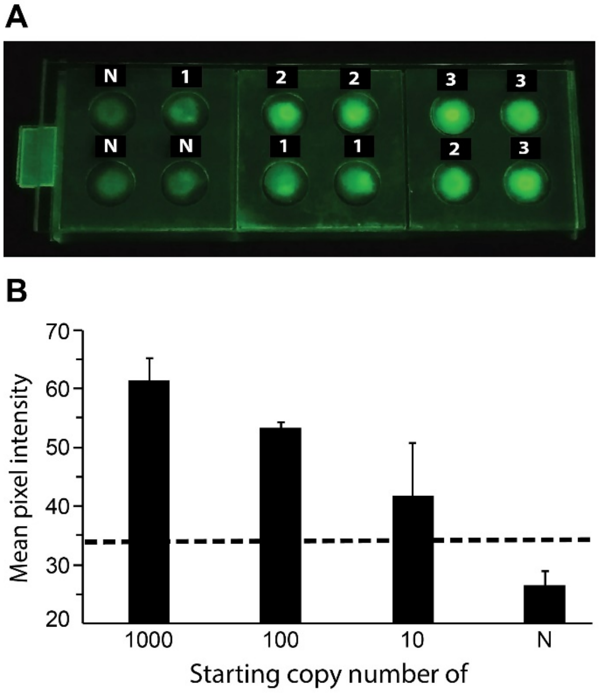


**Supplementary Figure S3**. Fluorescence imaging using optical filter. (A) End-point fluorescence detection. FLIPP-NAAT was imaged using a 510-550 nm band pass filter. (B) Mean green color intensities for different starting copy numbers of Mtb gDNA. The dashed line represents the threshold value of ($\mu_N$+3$\sigma_N$).

Fluorescence imaging was also performed using optical filters to check if filters helped in improving the imaging sensitivity. The mean intensities for each of the copy numbers was less than half of the intensities observed for imaging without the filters. While imaging without filters, it was seen that the mean intensities were co-related with the starting copy numbers with statistically significant differences. But for imaging through optical filters, the mean intensities for 100 and 10 copies were not statistically different. Though mean intensities for reactions with 1000 starting copies continued to be statistically higher than 100 copies and same was true for 10 starting copies in comparison with the negative control. Apart from non-specific interaction with single-

stranded DNA, the dye also seemed to have a component of green color even in its unbound state, which led to a prominent green signal in the negative controls when imaged through the filter.

**Supplementary Figure S4.** Optimization of the concentration of trehalose required for dry-storage of LAMP reagents

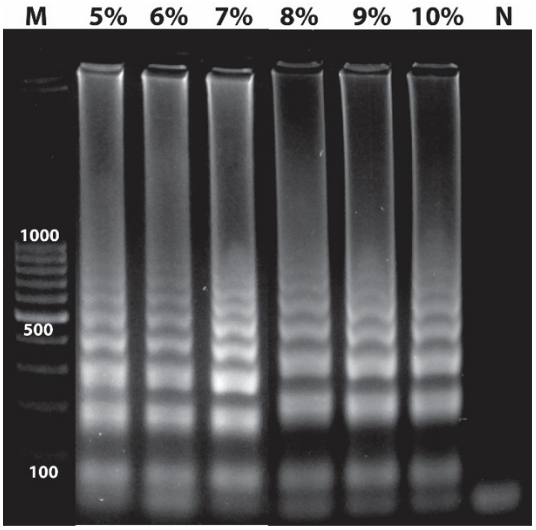


**Supplementary Figure S4**. Optimization of dry-storage of LAMP reagents. LAMP in tube was conducted with different concentrations of trehalose to check for the effect of trehalose on DNA amplification. LAMP amplicons were analysed using gel electrophoresis. The numbers on top of the wells indicate the percentage of trehalose used. M – DNA ladder, N – no template control.

**Supplementary Figure S5**. TB testing in FLIPP-NAAT using DNA extracted by conventional techniques

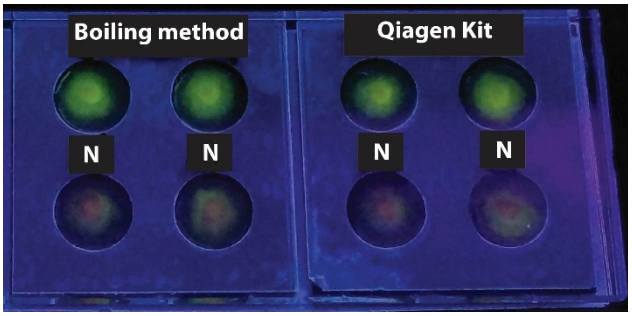


**Supplementary Figure S5**. LAMP from Mtb gDNA obtained by different DNA extraction methods. Mtb gDNA was extracted from the H37Ra cells using an in-house boiling method and Qiagen DNA extraction kit. The extracted DNA was used for DNA amplification in FLIPP-NAAT. Labels on the top of the reaction zones indicate the technique used for DNA extraction. N- no template control.

**Supplementary Figure S6. TB testing for clinical samples in FLIPP-NAAT**

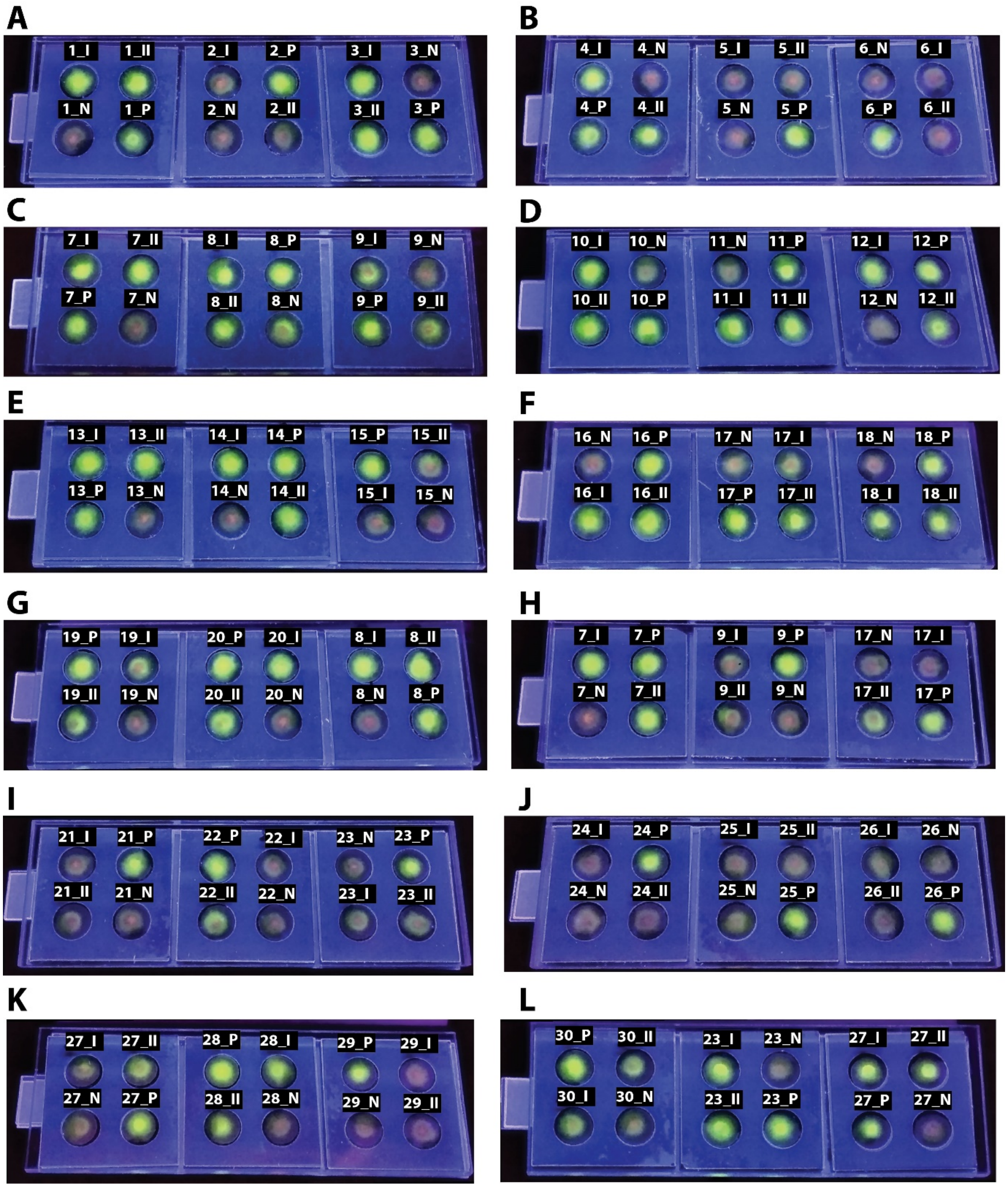


**Supplementary Figure S6**. TB testing in FLIPP-NAAT for clinical samples. 30 blind clinical samples were tested in FLIPP-NAAT and the results were captured using a cell phone camera. Numbers on top of each testing zone represent the sample number. Each sample was tested in duplicates and suffix ‘I’ and ‘II’ represent the duplicates for each sample. N – Negative control. P – Positive control.

The protocol for testing the twenty clinical samples in FLIPP-NAAT is described in the methods section of the main manuscript. Images were captured using a cell phone camera and intensity analysis was done in the green channel using ImageJ. Supplementary Figure S7 shows the end-point images for all the 12 devices used to test the 30 samples. Since one of the negative controls for the device in panel (C) (8_N) showed amplification, this device was declared as a failed test and samples 7, 8 and 9 were re-tested in devices as shown in panel G and H. Sample 17 was also tested twice (panel F and H) as in the first run (panel F), one of the duplicates showed amplification. This observation was consistent on repeating the test for sample 17 in panel H, where again only one of the duplicates showed amplification. All four of the sample 17 test results were also analyzed using gel electrophoresis to confirm the results from FLIPP-NAAT. The results were found to be consistent and are explained in the next section. Only one anomaly was found during testing of these 30 clinical samples, where sample 9 showed a positive test result in panel (C) but not in panel (H). Though device in panel (C) was deemed as a failed test, both the positive and negative controls in the module for sample 9 had worked perfectly fine. The ambiguity was resolved by analyzing the amplicons for sample 9 from device H using gel electrophoresis and the details are explained in the next section.

**Supplementary Figure S7. New protocol of TB testing for smear negative clinical samples in FLIPP-NAAT**

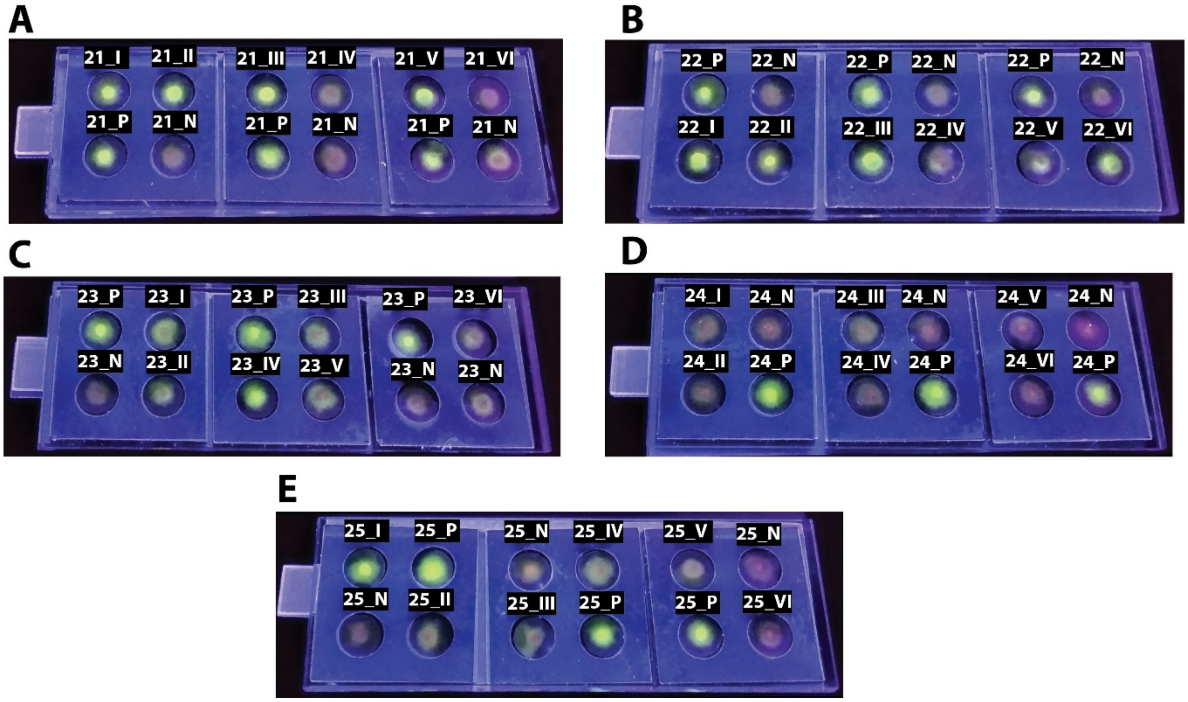


**Supplementary Figure S7**. TB testing in FLIPP-NAAT for lower bacillary load clinical samples. The five sputum smear microscopy negative clinical samples were tested in FLIPP-NAAT with six replicates per sample instead of duplicates and the results were captured using a cell phone camera. Numbers on top of each testing zone represent the sample number. Each sample was tested in duplicates and suffix 'I, II, III, IV, V and VI' represent the duplicates for each sample. N – Negative control. P – Positive control.

A loss in amplification was observed for sputum smear negative samples when the results for testing in FLIPP_NAAT (for duplicates per sample) were compared with the results from GeneXpert. This was attributed to the very small amount of starting template added to the reaction zones and the challenge was solved by running a greater number of replicates per sample. Six replicates were run per sample for the 5 sputum smear negative GeneXpert positive samples and the test was deemed to be positive if at least one out of the six replicates showed amplification.

**Supplementary Figure S8. Gel electrophoresis results for TB testing from clinical samples**

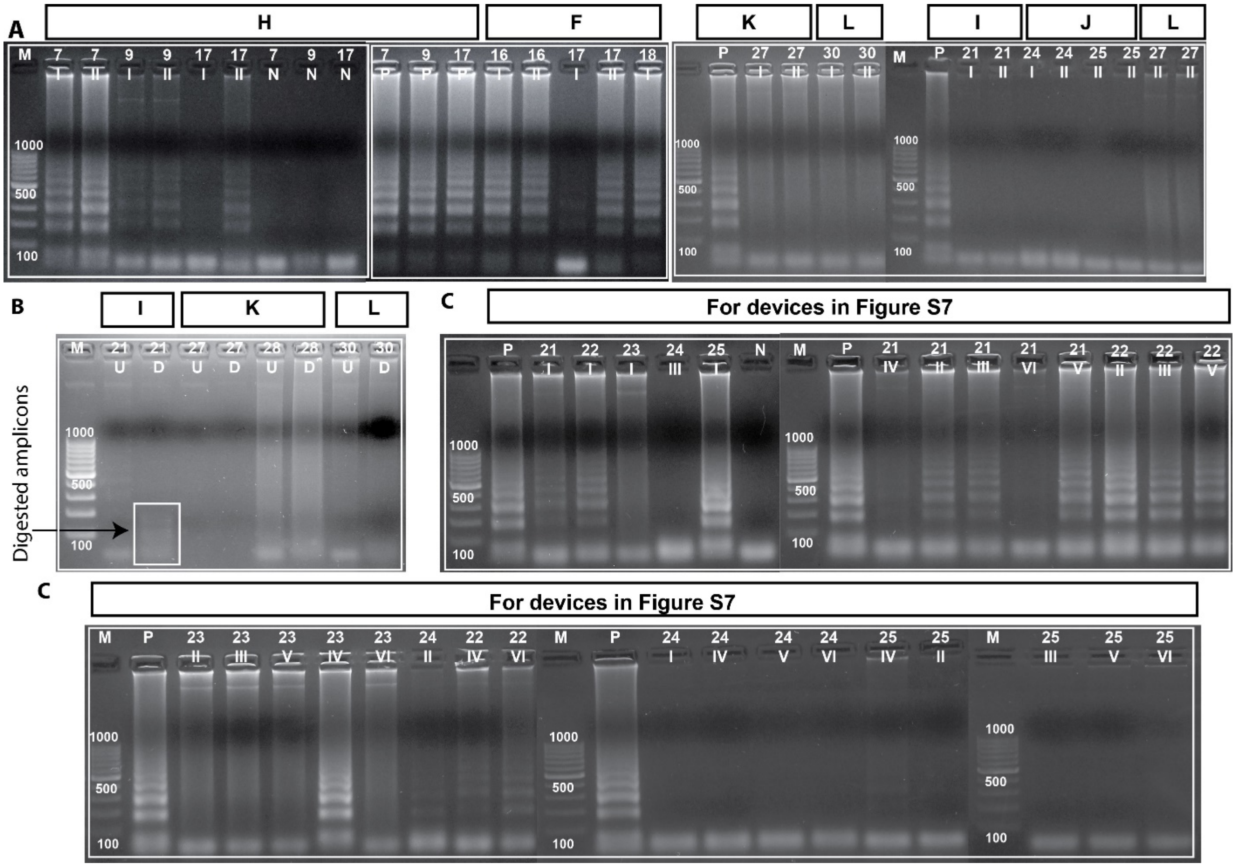


**Supplementary Figure S8**. Confirmation of LAMP patterns for amplification from clinical samples in FLIPP-NAAT. DNA amplification was performed in FLIPP-NAAT, amplicons were eluted from paper as explained in Fig. 5 in the main manuscript and the products were analysed using gel electrophoresis. The numbers on top of each well represent the sample number and suffix 'I, II, III, IV, V and VI' represent sample duplicates. M – DNA ladder, N – negative control, P – positive control, U- Undigested amplicons and D- digested amplicons. (A) Comparison of gel patterns for amplification from clinical samples to confirm the banding patterns. (B) Digestion of amplicons from clinical sample to confirm target-specific amplification. (C) Comparison of gel patterns for amplification from smear negative clinical samples to confirm the banding patterns. Alphabets on the top of gel lanes correspond to figure panels in Supplementary figure S6. The lanes marked within a white box are from the same gel and original gel images have been used.

LAMP amplicons from the reaction zones of the device from panel H, F, K, L, I, J and devices in Supplementary figure S8 were analyzed using gel electrophoresis. The gel patterns obtained for all the true positive clinical samples were identical to the positive controls which were set-up using 3.5 µl of 100copies/ µl of purified *Mtb* gNDA. However, the gel patterns for the false positive samples (Sample 27,28 and 30) we completely different from the target-specific

amplification pattern and the amplicons also did not get digested using the restriction enzyme *AatII*. All the negative controls did not show any amplification. Consistent with the FLIPP-NAAT results, sample 17_I in device H did not show any amplified products on the gel, while sample 17_I from device F showed very faint bands. This was also corroborated by the smear microscopy and GeneXpert results, both of which had classified sample 17 as weakly infected. Since one of the duplicates for sample 17 showed consistent amplification, sample 17 was considered as positive for FLIPP-NAAT test results.

Even though sample 9_I and 9_II from the device in panel H appear slightly green to the eye, the intensity from the test zones was not found to be higher than the threshold during the intensity analysis (Supplementary table S4). The gel results for sample 9_I and 9_II from device in panel H helped in resolving the ambiguity and confirming that sample 9 is a true positive.

**Supplementary Figure S9**. Uncropped images for gel electrophoresis analysis

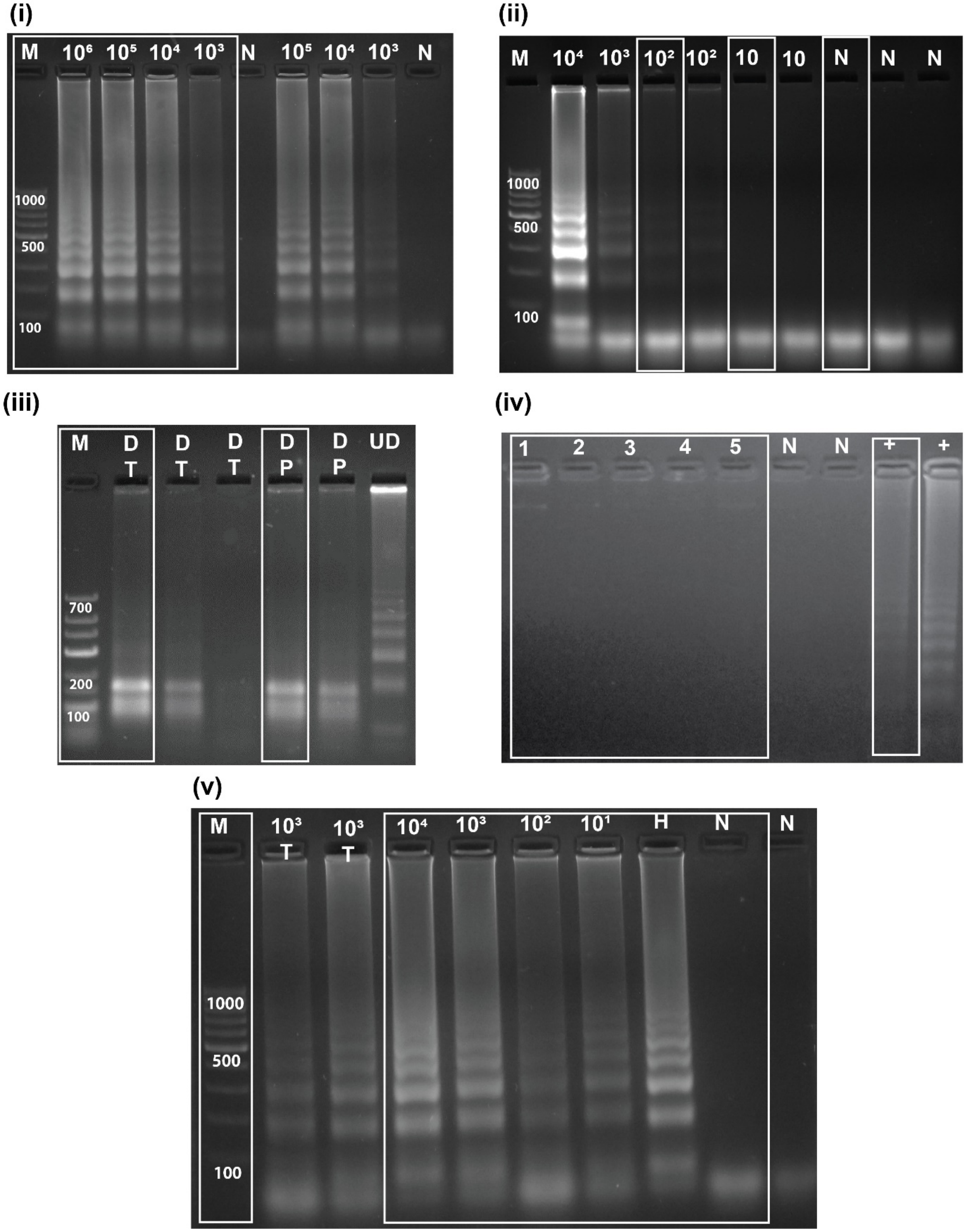


**Supplementary Figure S9**. Uncropped images for gel electrophoresis analysis. Sections marked within white boxes represent the lanes used in the figures for the main manuscript. **(i), (ii)** and **(iii)** LAMP in tube. Gel electrophoresis analysis of products of LAMP reactions conducted in tube with varying starting

copy numbers of Mtb gDNA ($10^6$copies up to 10 copies). The numbers on top of the lanes indicate the starting copy number of the template. N- No template control, M- DNA marker and D- digested LAMP amplicons. Reaction time was 40 minutes. **(iv)** Specificity of the LAMP assay tested against non-Mtb targets. +: Positive control (Mtb gDNA), 1: plasmid with hepatitis C insert, 2: plasmid with dengue insert, 3: E. coli gDNA, 4: Human gDNA, 5: Mycobacterium smegmatis gDNA. LAMP amplification in paper. **(v)** Gel electrophoresis analysis of products of LAMP reactions conducted in paper starting from varying number of starting Mtb gDNA copies ($10^4$ up to 10). The numbers on top of the wells indicate the starting copy number of the template. H- 1000 copies of human gDNA were added to the reaction mix along with 1000 copies of Mtb gDNA; N- No template control; M- DNA marker; and D- digested LAMP amplicons. T- LAMP reaction in tube and P- LAMP reaction in paper. The reaction time was 40 minutes.

## Supplementary Methods

<u>Extraction of DNA from Mtb cultures</u>

DNA obtained from Mtb bacterial cultures (strain H37Ra) was extracted using two methods: i) QIAamp DNA mini kit (Qiagen, 51304), and ii) an in-house crude boiling method. In the former, Mtb cells were lysed and DNA extracted following the protocol recommended by the manufacturer; 1 µl of the solution collected at the end of the extraction protocol was directly added to the LAMP mix. In the latter (in-house) method, 1ml of Mtb bacterial cell culture was centrifuged at 7000 rpm for 5 minutes to pellet down the bacterial cells. The supernatant was removed, and the pellet was resuspended in 200 µl TE buffer (10mM Tris base, 1mM EDTA). The resulting suspension was vortexed and kept at 95˚C for 30 minutes to lyse the cells. The turbid solution obtained was centrifuged at 8000 rpm for 5 minutes and 1 µl of the supernatant was directly added to the LAMP mix.

## Supplementary Notes

### Supplementary Note S1. Why LAMP?

While several isothermal nucleic acid amplification techniques exist, we chose LAMP for FLIPP-NAAT because of its multiple distinctive characteristics, e.g. i) enhanced specificity because of

the need of at least 4 primers, ii) an operating temperature range of 60-65˚C that is far from ambient, reducing baseline enzyme activity during storage, and iii) it's non-proprietary nature that enables acquiring individual reaction components from different vendors, providing flexibility for cost reduction. There are, however, certain challenges associated with LAMP. Primer dimer formation in LAMP leads to ladder-like patterns on gels reminiscent of target-specific amplification. Target-specific and non-specific amplification can be distinguished only by conducting enzyme digestion of products. The larger challenge with LAMP, however, is that because the number of amplicons produced is high, the method is prone to carryover amplicon contamination via aerosols. Post-amplification analysis was always conducted in a separate laboratory from the one in which LAMP reactions were set-up to avoid carryover contamination.

**Supplementary Note S2. Comparison of FLIPP-NAAAT with existing similar designs**

The device design by Seok et al.[1] and Ahn et al.[2] consisted of an assembly of polysulphone membrane, wax patterned polyethersulphone membrane, and glass fiber; physically stacked one above another and covered with ELISA sealing tape. The assembly was placed in a petri dish containing moist toilet tissues to maintain humidity and the petri dish was sealed using an aluminum tape. Nucleic acid amplification was demonstrated using LAMP and recombinase polymerase amplification (RPA). Fluorescence measurements were recorded at 10-min intervals by taking the set-up out of the oven and it was placed back at 63 °C in the oven immediately after measurement. The device from Trinh and Lee[3] consisted of a CNC milled pattern on a middle polycarbonate layer sandwiched between two sealing films. The chip was centrifuged after sample addition to distribute the sample to the respective reaction zones. Both the studies used fluorescence-based detection with imaging done in a high-end Bio-Rad Molecular Imager Gel ChemiDoc. Both the designs had multiple user steps, required ancillary equipment, and

fluorescence imaging was done in an expensive set-up. FLIPP-NAAT, on the other hand, can contain all the components required for NAATs within the device and the user experience is very simple and minimal. One of the most desirable features of FLIPP NAAT is that the material cost of making a 12-reaction-zone device is only $0.88 and the cost of reagents required per reaction zone is $0.43 (see Supplementary Table S3 and S4 online). Modular design is a powerful feature of FLIPP-NAAT and using the current definition of a module, devices may be designed to contain 4N reaction zones (where N = 1, 2, 3…), without any major modification in fabrication methods. The device design is a simple layer-by-layer assembly of paper pads, plastics, and adhesives, and therefore it is compatible with mass manufacturing using injection molded plastic components. The very low-cost imaging box designed for cell phone-based filter-free fluorescence imaging is also an important development for enabling point-of-care NAATs.

**Supplementary Note S3. Optimizing FLIPP-NAAT design**

Several design challenges were overcome to develop the final FLIPP-NAAT prototype. Because the paper reaction zones in FLIPP-NAAT are in close proximity to each other, cross contamination was a challenge. The device dimensions were modified to provide more area of contact for the PSA to ensure it did not detach during heating at 63˚C. A vice was used to pressurize the device after fabrication to ensure that all layers were tightly secured. In the current design, there exists a 1-mm gap between modules, where there is discontinuity in both the top black acrylic covers and the bottom layer of PSA. The sudden change in surface properties from PSA to acrylic in these gaps helps in avoiding spread of leaking fluid, if any. Different types of materials were iterated to determine the most suitable material for each layer to optimize fluorescence detection. Transparent acrylic bottom allowed imaging from the bottom. Black acrylic covers, PSA, and the transparency cover gave minimum background fluorescence. The entirely plastic assembly of the device (i.e. no

use of glass) made it amenable to pressurized sealing, which created evaporation-free reaction zones and avoided cross-talk between the reaction zones.

**References:**


1. Seok, Y. *et al.* A Paper-Based Device for Performing Loop-Mediated Isothermal Amplification with Real-Time Simultaneous Detection of Multiple DNA Targets. *Theranostics* **7**, 2220–2230 (2017).
2. Ahn, H., Batule, B. S., Seok, Y. & Kim, M. G. Single-Step Recombinase Polymerase Amplification Assay Based on a Paper Chip for Simultaneous Detection of Multiple Foodborne Pathogens. *Anal. Chem.* **90**, 10211–10216 (2018).
3. Trinh, T. N. D. & Lee, N. Y. A rapid and eco-friendly isothermal amplification microdevice for multiplex detection of foodborne pathogens. *Lab Chip* **18**, 2369–2377 (2018).
